# The Arab Countries’ Contribution to the Research of Neurodegenerative Disorders

**DOI:** 10.7759/cureus.17589

**Published:** 2021-08-31

**Authors:** Jad El Masri, Razan Dankar, Diala El Masri, Hani Chanbour, Said El Hage, Pascale Salameh

**Affiliations:** 1 Medicine, Lebanese University Faculty of Medicine, Beirut, LBN; 2 Medicine, University of Balamand Faculty of Medicine, Beirut, LBN; 3 Epidemiology, Lebanese University Faculty of Medicine, Beirut, LBN; 4 Department of Primary Care and Population Health, University of Nicosia Medical School, Nicosia, CYP; 5 Toxicologie Clinique et Toxicologie (INSPECT-LB) - Liban, Institut National de Santé Publique, Beirut, LBN

**Keywords:** bibliometric analysis, research productivity, arab world, neurodegenerative diseases, alzheimer’s, parkinson

## Abstract

Background: Neurodegenerative diseases are disorders in which nerve cells start to lose function due to different causes. Like many other illnesses, they are considered to be highly prevalent in the 22 Arabic-speaking countries known to constitute the Arab world. The two most prevalent neurodegenerative disorders are Alzheimer's disease and Parkinson's disease.

Aim: The aim of this paper is to assess the amount of research dedicated to neurodegenerative diseases by the Arab countries during a 15-year period, between 2005 and 2019.

Methods: The number of publications by each Arab country as well as some non-Arab speaking countries was retrieved from PubMed. Publications in top 10 neuroscience journals were also tracked using the same method with each journal’s name included. The numbers were then normalized with respect to the average population and average gross domestic product (GDP) in each country to eliminate bias.

Results: Arab countries were shown to contribute only 1,311 (0.774%) of the 169,330 articles published worldwide on neurodegenerative disorders. These 1,311 also constitute only 0.660% of the 198,869 Arab publications during the indicated period. Saudi Arabia had the highest contribution to these numbers with more than one-quarter the number of publications on neurodegenerative disorders. Approximately one-third of all neurodegenerative disease-related articles were associated with Alzheimer's disease, whereas one-fifth were related to Parkinson's disease. For the top 10 neuroscience journals, only a minimal contribution by Arab countries was noted.

Conclusion: Although an increase in the number of articles by the Arab world was noted from 2013 onward, the contribution of the Arab countries on the subject to the number of publications still seems to be insufficient.

## Introduction

The Arab world consists of 22 Arabic-speaking countries, concentrated mainly in the Middle East and North Africa. As in any part of the world, the various Arab countries differ significantly with respect to incomes, where some are considered high-income countries and others low-income. Although the amount of research conducted by the Arab world has increased remarkably during the last few years, Arab countries still seem to provide a deficient contribution to the total research conducted worldwide [1]. Moreover, the fact that this contribution is concentrated in a few of all 22 Arab countries intensifies the problem. Thus, in a study by El Rassi et al., only four countries (Qatar, Tunisia, Lebanon, and Kuwait) contributed more papers per million people than the world average [1].

Neurodegenerative disorders are diseases where nerve cells start to lose function and consequently die, and proteins accumulate in the brain and other peripheral organs [2]. They are mainly classified according to the deposited protein and the better-known tauopathies, alpha-synucleinopathies, prion diseases, trinucleotide repeat diseases, neuroserpinopathy, ferritinopathy, and cerebral amyloidoses [2]. Neurodegenerative disorders are an emerging cause of mortality worldwide, many of which affect the elderly [3]. Globally, Alzheimer’s disease is considered to be the most prevalent neurodegenerative disorder constituting 60-80% of all dementia cases, with approximately 24 million people affected with the disease worldwide [4]. Parkinson’s disease ranks second after Alzheimer's disease as to prevalence, affecting an estimated 0.3% of the total human population [4].

Studies on Arab countries proved neurodegenerative diseases to be prevalent regionally, ranging between 1.1-2.3% in the ≥ 50 years population and 13.5-18.5% in the ≥ 80 years population [5]. Many factors have been proven to contribute to the incidence of neurodegenerative diseases, including age, education, gender, genetic factors, and health conditions [5]. It is noteworthy that studies have shown that hypertension and type-2 diabetes mellitus facilitate the onset of neurodegenerative disorders through neuroinflammation [6], which explains one cause of the high prevalence in Arab countries. For instance, Arab countries have a high prevalence of hypertension (29.5%), which is higher than in many other regions [7]. Similarly, type-2 diabetes was shown to be highly prevalent in several of the 22 Arabic-speaking countries [8].

Keeping in mind the high incidence of neurodegenerative disorders and their risk factors, in addition to the low quantity of research on these disorders in the Arab world, we performed a bibliometric analysis of these countries’ contribution to such research. Therefore, this paper aims to assess the number of publications concerning neurodegenerative diseases between 2005 and 2019 from the 22 Arab countries, focusing mainly on the two most prevalent disorders: Alzheimer's disease and Parkinson's disease.

## Materials and methods

Database and search strategy 

A screening was done for neurodegenerative-related publications for a period of 15 years (2005-2019) from all 22 Arab countries, constituting Algeria, Bahrain, Comoros, Djibouti, Egypt, Iraq, Jordan, Kuwait, Lebanon, Libya, Mauritania, Morocco, Oman, Palestine, Qatar, Saudi Arabia, Somalia, Sudan, Syria, Tunisia, United Arab Emirates, and Yemen.

Using Boolean operators AND, OR, and NOT, we searched PubMed for “neurodegenerative diseases”, “Alzheimer’s disease”, and “Parkinson’s disease”; the Medical Subject Headings (MeSH) for “name of Arab country required” affiliation, and the Mesh Dates "2005 - 2019". Cities in the United States (US) called Lebanon were excluded, and in Palestine, we used West Bank and Gaza.

Countries with the highest research productivity related to neuroscience were also searched, including the US, the United Kingdom (UK), Germany, Japan, Canada, Italy, France, China, Netherlands, and Australia [9].

The same methods were used to track publications in the top 10 neuroscience journals, searching for the journal’s name in addition to the procedures stated above.

Inclusion and exclusion criteria

All types of articles were included, as long as they had an author from the targeted country, and the article was published between 2005 and 2019.

Interpretation and comparison

The average population and gross domestic product (GDP) were acquired for each country between 2005 and 2019 using data from the 2019 World Population Prospects and the 2019 World Bank report on GDP respectively [10-11]. For each country, the number of publications per 1,000,000 persons was calculated, as well as per GDP. Similar approaches have been used in other bibliometric analyses [12-14].

Data were analyzed using IBM SPSS Statistics for Windows, version 22.0, released 2013 (IBM Corp, Armonk, NY) where linear regression was used to relate the number of publications to both the average population and the average GDP.

## Results

Table 1 represents the number of articles published by Arab countries on neurodegenerative diseases during a 15-year period, between 2005 and 2019. With 1,311 publications on neurodegenerative diseases, the Arab world contributed 0.774% of the 169,330 studies conducted worldwide concerning this issue. This represents 0.66% of the 198,869 total papers published by the Arab world. Among the 22 Arab countries studied, three namely Comoros, Djibouti, and Somalia, had no publications on neurodegenerative diseases during this period. Saudi Arabia had the highest number of neurodegenerative disease-related articles (357) accounting for 27.23% or more than one-quarter of the total published by Arab countries. Egypt ranked second with 290 articles (22.1%), and Tunisia ranked third with 127 articles (9.7%). However, when studying the number of neurodegenerative disease-related articles with respect to the total number of articles published by a country, Mauritania had the highest percentage of 2.778% followed by Oman (1.282%).

**Table 1:** Number of neurodegenerative disease publications by Arab countries during a 15-year period, between 2005 and 2019

Country	Number of neurodegenerative diseases publications	Number of total publications	% Neurodegenerative publications of total
Algeria	38	4,062	0.935
Bahrain	3	1,460	0.205
Comoros	0	39	0.000
Djibouti	0	97	0.000
Egypt	290	53,290	0.544
Iraq	11	4,407	0.250
Jordan	67	10,817	0.619
Kuwait	23	5,646	0.407
Lebanon	70	12,227	0.573
Libya	10	997	1.003
Mauritania	3	108	2.778
Morocco	72	7,618	0.945
Oman	62	4,835	1.282
Palestine	4	1,042	0.384
Qatar	82	8,265	0.992
Saudi Arabia	357	53,898	0.662
Somalia	0	94	0.000
Sudan	2	3,069	0.065
Syria	2	1,356	0.147
Tunisia	127	14,633	0.868
United Arab Emirates	83	9,731	0.853
Yemen	5	1,178	0.424
Total	1,311	198,869	0.659
Worldwide	169,330	13,995,404	1.210

**Figure 1:**
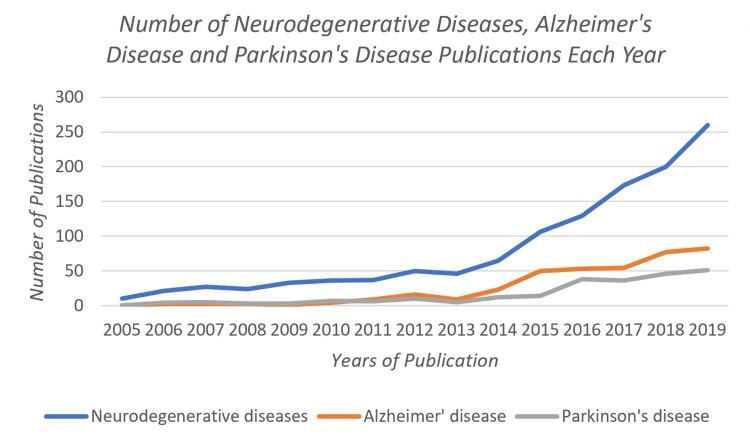
Number of neurodegenerative diseases, Alzheimer's disease, and Parkinson's disease publications each year between 2005-2019

A significant increase in the number of publications began in 2013 and, thus, in the last seven years, 933 articles were published accounting for 80%, 90%, and 84% of the total articles related to neurodegenerative diseases, Alzheimer’s disease, and Parkinson's disease, respectively. (Figure 1)

**Figure 2:**
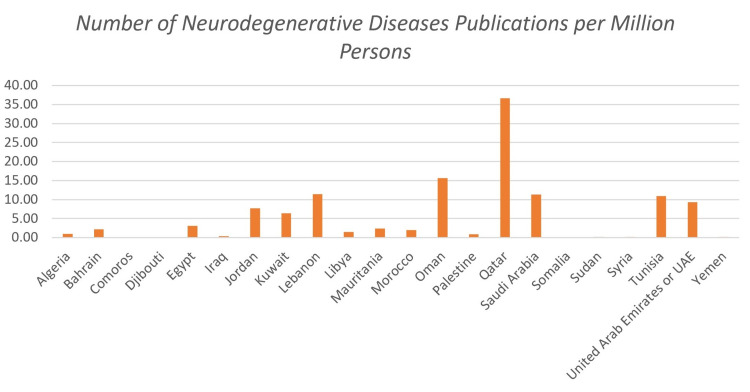
Number of neurodegenerative diseases papers published per million persons between 2005 - 2015 for each of the indicated countries

Figure 2 shows the number of publications concerning neurodegenerative disorders per million persons for each of the indicated countries for the 15-year duration. These results were normalized with respect to the average population size in order to reduce or eliminate any possibility of biased results. Qatar had the highest number of publications on neurodegenerative diseases per million persons (36.69). Second came Oman with 15.69 per million persons. Disregarding countries with no publications, Iraq and Yemen had the lowest numbers, 0.31 and 0.19 respectively. Linear regression suggested a weak correlation between the number of published articles and an average population (r = 0.328), where the average population explained 10.7% of publications regarding neurodegenerative diseases (r^2^ = 0.107).

**Figure 3:**
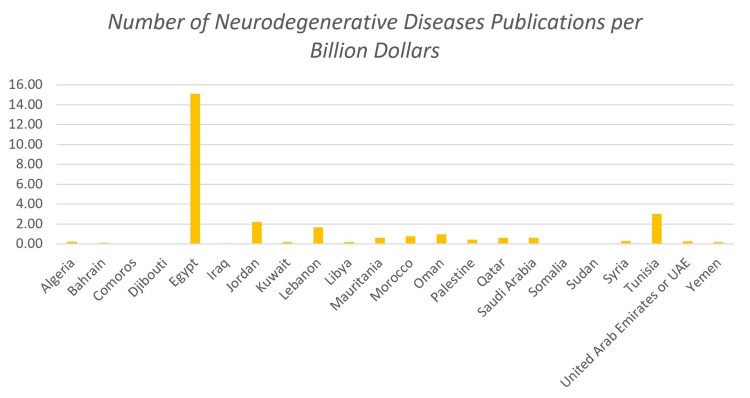
Number of neurodegenerative diseases publications per billion dollars for each of the indicated countries for the 15-year duration

Figure 3 shows the number of publications concerning neurodegenerative disorders per billion dollars for each of the indicated countries for the 15-year duration. These results were normalized with respect to the average GDP in order to reduce or eliminate any possibility of biased results. Egypt has a dominant gap with respect to other countries when it comes to the number of publications per billion dollars. This accounts for 15.1. Most countries have very low numbers of publications per billion dollars, with Bahrain having the least (0.1), other than those with zero publications. Linear regression showed a significant relationship between the number of published articles and GDP (p=0.003), where the correlation was strong (r=0.608), and GDP explains 39.6% of neurodegenerative disease-related publications (r^2^ = 0.396).

Neurodegenerative studies related to Alzheimer’s disease represented 422 out of 1,311 (32.891%) or approximately one-third of the total Arab neurodegenerative studies. Similar to the results regarding total neurodegenerative diseases, Saudi Arabia had the highest number of publications (130), followed by Egypt (103), together contributing to more than one-half of the Arab publications in this field.

Neurodegenerative studies related to Parkinson’s disease comprised 253 out of 1311 (19.298%), or approximately one-fifth or 422 out of the total of 1311 Arab neurodegenerative studies. Egypt had the highest number of publications (68), followed by Saudi Arabia (42).

**Table 2:** The number of Alzheimer's disease, Parkinson's disease, and neurodegenerative diseases publications

Country	Number of Alzheimer's disease publications	Number of Parkinson's disease publications	Number of neurodegenerative disease publications
Algeria	15	4	38
Bahrain	0	2	3
Comoros	0	0	0
Djibouti	0	0	0
Egypt	103	68	290
Iraq	8	1	11
Jordan	14	23	67
Kuwait	3	4	23
Lebanon	12	12	70
Libya	4	5	10
Mauritania	0	1	3
Morocco	15	10	72
Oman	22	12	62
Palestine	0	1	4
Qatar	23	29	82
Saudi Arabia	130	44	357
Somalia	0	0	0
Sudan	1	0	2
Syria	1	0	2
Tunisia	35	15	127
United Arab Emirates	33	22	83
Yemen	3	0	5
Total	422	253	1311
Worldwide	56,813	38,498	169,330

Table 3 shows that the US leads the world in the field of research related to neurodegenerative diseases, having approximately 40,000 publications. The UK follows with 14,241 publications or approximately one-third the number of the US. Regarding the percentage of total publications, Italy is the first having 2.325% of the publications related to neurodegenerative diseases.

**Table 3:** Neurodegenerative diseases publications between 2005 and 2019 in countries having the highest neuroscience-related research work *total publications = non-neurodegenerative + neurodegenerative diseases publications

Country	Number of publications on neurodegenerative diseases	Total number of publications*	Percentage of neurodegenerative diseases publications of the total publications*
USA	40,242	2,827,978	1.423
United Kingdom	14,241	882,586	1.614
China	11,581	1,363,499	0.849
Italy	11,452	492,648	2.325
Germany	10,048	664,330	1.513
Japan	8,988	677,246	1.327
France	7,407	461,782	1.604
Canada	6,547	465,468	1.407
Australia	5,035	389,891	1.291
Netherlands	4,485	300,045	1.495

Table 4 shows that the Arab world published only 150 papers in the 10 top-ranked neurology journals, and even fewer (21 papers) were neurodegenerative diseases publications. *Neurology* published the majority of Arab studies, i.e. 73 publications.

**Table 4:** Comparison between the number of neurodegenerative diseases-related publications from Arab countries published between 2005-2012 and 2012-2019, in the top 10 neurology journals, per their respective 2019 impact factors (IFs) *Total = neurodegenerative diseases-related + non-neurodegenerative diseases-related publications 2005 - 2019

Journal	IF	Neurodegenerative diseases-related publications		Total publications*
		2005 - 2012	2012 - 2019	
Neuron	14.45	0	0	4
Nature Neuroscience	20.07	0	1	2
The Lancet Neurology	30.04	0	1	4
Nature Reviews Neuroscience	33.65	0	0	1
Neuroimage	5.90	0	0	8
Neurology	8.77	4	9	73
Journal of Neuroscience	5.68	0	1	25
Brain	11.33	1	4	22
Neuroscience & Biobehavioral Reviews	8.33	0	0	8
Nature Reviews. Neurology	27.00	0	1	3

## Discussion

In this bibliometric analysis, we assessed the neurodegenerative disease research productivity in all 22 Arab countries over a 15-year period, ranging from 2005 till 2019. As our results indicated, Saudi Arabia followed by Egypt published the greatest number of neurodegenerative diseases-related articles during this timeframe. In similar analytic studies, Saudi Arabia and Egypt have also ranked the highest in the number of Arab world publications on tobacco use [15] and biomedical topics [16]. Saudi Arabia also published the most among all Arab countries on mental health research topics [17] and substance use disorders [18].

Nevertheless, it was essential to normalize this by using some parameters like population size and GDP. Among the two indicators used, we found GDP to be more representative in comparing the Arab countries' neurodegeneration research activity, with a strongly positive correlation. Egypt was ranked first among the 22 Arab countries in number of publications per GDP. Population size, however, which showed that Qatar had the highest number of neurodegenerative diseases publications per million persons, had a weaker correlation.

We can therefore say that GDP, or the number of publications per billion dollars, is the most reliable marker for neurodegenerative diseases publications in the Arab world. Similarly, GDP has been found to be the most reliable indicator for stroke publications [19] and for biomedical research in Arab countries [16]. In contrast, for psoriasis-related publishing activity in the Arab world, population size was considered the most accurate marker [20].

Our results revealed that the number of neurodegeneration-related publications in Arab countries has been on the rise in the past seven years. One cannot, however, overlook the fact that the total number of neurodegenerative publications in the Arab world account for only 0.77% of the research done worldwide on this subject in the past 15 years. In fact, several studies have addressed the problem of shortage of medical research in Arab countries, proving that they are still lagging behind in biomedical publications, average citation frequency, and top journal publishing activity [21,1]. A bibliometric analysis, done between the years 1996 and 2012, indicated that the Arab world publications represented only 4% of those published by the USA [1].

This inadequacy of medical publications in Arab countries can be traced to various factors including limited research facilities, inadequate academic advancements, and the absence of interest in research programs and funding opportunities [1]. The lack of political and economic stability also plays a major role in hindering research programs [22]. Arab countries are chronically suffering from corruption, lack of proper democracy, wars and internal conflicts, which are all quite unfavorable for proper economic growth [23]. This can actually be illustrated by the fact that most Arabian countries belong to low-income economic groups, aside from six Arabian Gulf countries: Saudi Arabia, Bahrain, Kuwait, Qatar, United Arab Emirates, and Oman [24]. The poor economic resources might also help explain our results that proved GDP to be the most crucial factor in assessing neurodegenerative diseases research in Arab countries.

Furthermore, in our analysis, it was evident that Alzheimer’s disease-related publications made up one-third of the total neurodegenerative disease studies in the Arab world. This might be justified by the importance of this inevitably progressive disease that has a major negative impact on the life of the patient’s family [25]. Another important aspect is the important prevalence of this neurocognitive disease. A systematic review done in 2019 to evaluate the epidemiology of dementia and Alzheimer’s disease in Arab countries revealed that the prevalence is indeed high, ranging between 13.5% and 18.5% among elderly patients > 80 years of age. Of those 50 years and older, the prevalence ranged between 1.1% and 2.3% [5]. Moreover, the WHO has reported dementia as a public healthcare priority, estimating its worldwide prevalence at 35.6 million [26]. Despite all this, research done in the Arab world on this subject remains scarce. It is encouraging to note that publications on this topic have been increasing in the last few years.

When comparing the number of neurodegenerative-related publications in countries with the highest research activity in this field to the combined Arab countries, a huge difference is present between the 10th country Netherland, which is around four times the sum of Arab publications. This difference becomes surprisingly enormous when compared to the 1st country USA, which is around 40 times the sum of Arab publications.

Regarding the quality of Arab papers in the field of neurodegenerative diseases, they were shown to be of low quality, since only 21 papers were published in top-ranked neuroscience journals, comprising no more than 2% of published neurodegenerative-related studies.

Limitations

The major limitation of this study was that only the PubMed database was searched, as the tracked number of publications might not be accurate. Furthermore, this study lacks publications written in languages other than English, such as French, which is widely used in some Arab regions, including Algeria, Comoros, Djibouti, Mauritania, Morocco, and Tunisia. Moreover, inaccuracy of data tracking may have occurred due to searching for the indexed and not the published date.

## Conclusions

To our knowledge, this is the first bibliometric analysis to assess research activity related to neurodegenerative diseases in the Arab world. Despite showing an increase in research productivity in the last few years, numbers remain far behind top-ranked countries in neuroscience research productivity. The low quality of research and strong correlation to GDP highlight the importance of collaboration between high- and low-income countries to establish new institutions that can come up with high-quality research.
